# An Efficient Synthesis of Novel Dispirooxindole Derivatives via One-Pot Three-Component 1,3-Dipolar Cycloaddition Reactions

**DOI:** 10.3390/molecules171112704

**Published:** 2012-10-26

**Authors:** Zhibin Huang, Qian Zhao, Gang Chen, Huiyuan Wang, Wei Lin, Lexing Xu, Hongtao Liu, Juxian Wang, Daqing Shi, Yucheng Wang

**Affiliations:** 1 Key Laboratory of Organic Synthesis of Jiangsu Province, College of Chemistry, Chemical Engineering and Materials Science, Soochow University, Suzhou 215123, China; 2 Tianjin ShuiGe Hospital, Tianjin 300120, China; 3 Zhejiang Medicine Co., Ltd. Xinchang Pharmaceutical Factory, Xinchang 312500, China; 4 Institute of Medicinal Biotechnology, Chinese Academy of Medical Sciences and Peking Union Medical College, Beijing 100050, China

**Keywords:** dispirooxindole, three-component reaction, 1,3-dipolar cycloaddition, azomethine ylide

## Abstract

A series of novel dispirooxindoles have been synthesized through three-component 1,3-dipolar cycloaddition of azomethine ylides generated *in situ* by the decarboxylative condensation of isatin and an *α*-amino acid with the dipolarophile 5-benzylidene-1,3-dimethylpyrimidine-2,4,6-trione. This method has the advantages of mild reaction conditions, high atom economy, excellent yields, and high regio- and stereo-selectivity.

## 1. Introduction

In recent decades, multicomponent reactions (MCRs) have emerged as a powerful synthetic strategy due to their efficiency, atom economy, high selectivity and convenience in the construction of multiple new bonds, which permit a rapid access to combinatorial libraries of complex organic molecules for efficient lead structure identification and optimization in drug discovery [1,2,3,4]. According to this method, the products are formed in a single step and diversity can be achieved simply by varying the reacting components. 

1,3-Dipolar cycloaddition of azomethine ylides with olefinic and acetylenic dipolarophiles has gained significance as it proceeds with high regiochemical and stereochemical selectivity yielding pyrroline and pyrrolidine derivatives [5,6,7], which are prevalent in a variety of biologically active compounds [8] and are also inhibitors of many diseases such as diabetes [9], cancer [10] and viral infections [11]. Because of their remarkable biological activities, significant efforts have been devoted to the synthesis of their novel derivatives.

Among the various nitrogen-containing heterocycles, functionalized pyrrolidine, pyrrolizidine and oxindole alkaloids have become important synthetic targets as they constitute classes of compounds with significant biological activity [12]. The synthesis of spiro compounds has drawn considerable attention of chemists as have their highly pronounced biological properties [13,14]. The spirooxindole system as the core structure of many pharmacological agents and natural alkaloids [15,16,17,18], and has potent nonpeptide p53-MDM2 inhibitory activity [19]. Elacomine, spirotryprostatins A and B are some of the alkaloids containing spiropyrrolidinyloxindole ring systems. Some spiropyrrolidines are potential antileukaemic and anticonvulsant agents [20] and possess antiviral and local anaesthetic activities [21].

Barbituric acid has widely been used in the manufacture of plastics [22], textiles [23], polymers [24] and pharmaceuticals [25,26,27,28]. Barbiturates (derivatives of barbituric acid) like pentobarbital and phenobarbital were long used as anxiolytics and hypnotics. Spirobarbiturates are a class of compounds with interesting pharmacological and physiological activity [29,30,31]. We have recently reported the regio- and stereoselective synthesis of novel dispirooxindole derivatives via multicomponent reactions [32,33,34,35,36]. To expand our research program which aims to synthesize new spiro compounds and nitrogen heterocycles with biological activities, we report herein, the efficient synthesis of a series of novel dispirooxindole derivatives in excellent yields by the three-component 1,3-dipolar cycloaddition reaction of nonstabilized azomethine ylides generated *in situ* by the decarboxylative condensation of isatin and *α*-amino acids with 5-benzylidene-1,3-dimethylpyrimidine-2,4,6-trione using ethanol under reflux conditions.

## 2. Results and Discussion

**Scheme 1:**
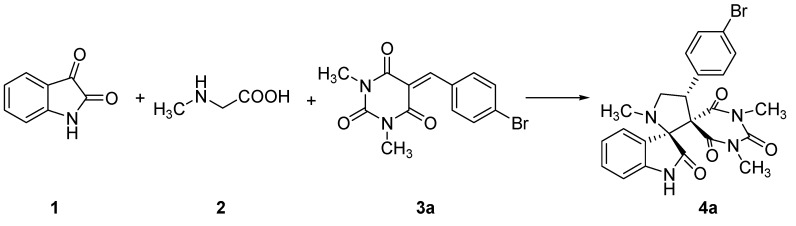
The model reaction.

In an effort to optimize this process, the three-component reaction of isatin (**1**), sarcosine (**2**), and the dipolarophile 5-(4-bromobenzylidene)-1,3-dimethylpyrimidine-2,4,6-trione (**3a**) was carried out in various solvents under reflux conditions as a simple model reaction in order to determine the best reaction solvent (Scheme 1). The results are summarized in Table 1. As can be seen from the data, the reaction could be efficiently carried out in solvents such as ethanol, methanol, acetonitrile, THF and 1,4-dioxane. In particular, the reaction using ethanol as the solvent resulted in higher yields and shorter reaction times than those using methanol, acetonitrile, THF and 1,4-dioxane. Thus, ethanol, which is a low cost bio-renewable product with low toxicity to human health and is relatively non-hazardous to the environment was chosen as the solvent for all further reactions (Table 1, entry 1) [37].

**Table 1:** Optimization of solvent effect on the model reaction ^a^.

Entry	Solvent	Time (h)	Yield ^b^ (%)
1	Ethanol	2	84
2	Methanol	2	56
3	Acetonitrile	3	75
4	Tetrahydrofuran (THF)	6	80
5	1,4-Dioxane	8	60

^a^ Reaction conditions: isatin (0.5 mmol), sarcosine (0.5 mmol) and 5-(4-bromobenzylidene)-1,3-dimethylpyrimidine-2,4,6-trione (0.5 mmol) in solvent (10 mL) at reflux temperature; ^b^ Yields of isolated products.

Using the optimized reaction conditions, various structurally diverse 5-benzylidene-1,3-dimethyl-pyrimidine-2,4,6-triones were investigated (Table 2). It was found that the aromatic rings bearing either electron-withdrawing or electron-donating functional groups were suitable for the reaction, while the cycloaddition reactions with dipolarophiles carrying electron-donating substituents required a longer times and the yield decreased (Table 2, entry 2).

**Table 2:** Synthesis of dispirooxindole derivatives **4** via three-component reaction. 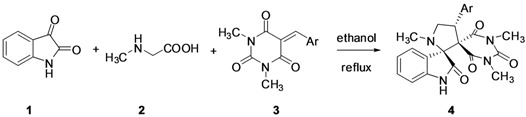

Entry	Product	Ar	Time (h)	Yield (%)
1	**4a**	4-BrC_6_H_4_	2	84
2	**4b**	4-CH_3_C_6_H_4_	2.5	75
3	**4c**	4-NO_2_C_6_H_4_	1.5	90
4	**4d**	4-ClC_6_H_4_	2	88

In order to establish the scope of this cycloaddition reaction, we extended the same protocol using istain (**1**), L-thioproline (**5**) and dipolarophiles **3** under the same reaction conditions to give a series of cycloadducts **6** in excellent yields (Table 3).

**Table 3:** Synthesis of dispirooxindole derivatives **6** via three-component reactions. 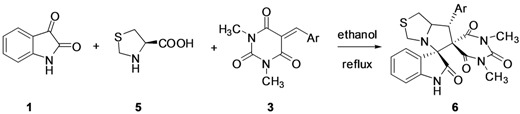

Entry	Product	Ar	Time (h)	Yield (%)
1	**6a**	4-BrC_6_H_4_	1	84
2	**6b**	4-CH_3_C_6_H_4_	2	81
3	**6c**	4-NO_2_C_6_H_4_	1	87
4	**6d**	4-ClC_6_H_4_	1	83
5	**6e**	C_6_H_5_	1	82
6	**6f**	2-NO_2_C_6_H_4_	1.5	82
7	**6g**	3,4-Cl_2_C_6_H_3_	1.5	88
8	**6h**	Thiophen-2-yl	3	86

**Figure 1:**
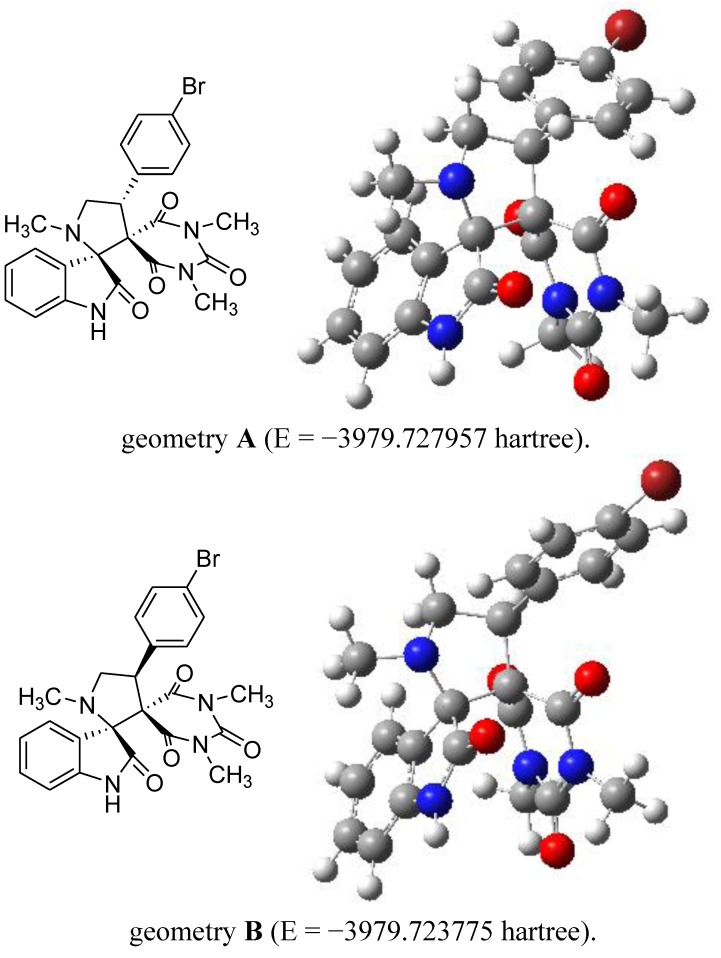
Optimized geometry of **4a**.

With the use of Discrete Fourier Transformation (DFT) and the B3LYP/6-31G computer programme [38], a geometrical optimization of product **4a** was obtained and is shown in Figure 1. From Figure 1, we found that the geometry **A** (*trans*) was more stable than geometry B (*cis*) (∆E = 10.98 kJ/mol).

To expand the scope of the current method, acenaphthenequinone (**7**) was examined as a replacement for isatin (**1**). The desired products **8** were obtained under the optimized conditions. The results are summarized in Table 4.

**Table 4:** Synthesis of dispirooxindole derivatives **8** via three-component reaction. 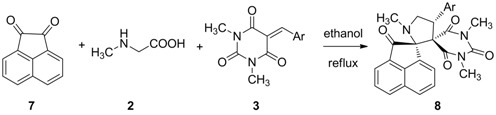

Entry	Product	Ar	Time (h)	Yield (%)
1	**8a**	C_6_H_5_	1	82
2	**8b**	3,4-Cl_3_C_6_H_3_	1.5	80
3	**8c**	3,4-OCH_2_OC_6_H_3_	3	78

**Figure 2:**
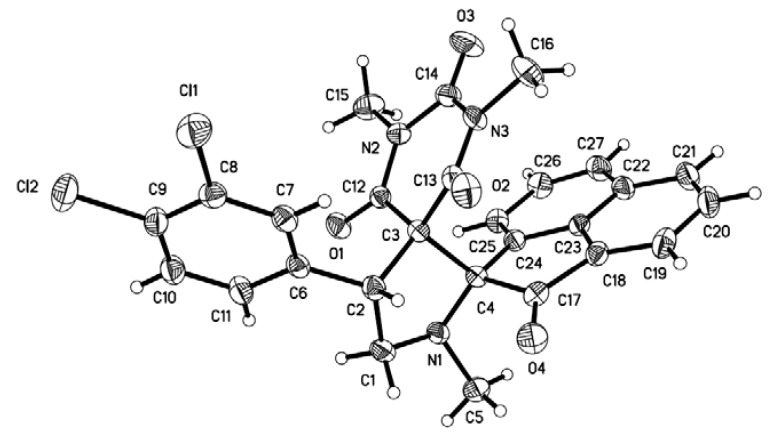
X-Ray crystal structure of compound **8b**.

The structures of the products were identified by IR, ^1^H-NMR, ^13^C-NMR and HRMS spectra. The structure and regiochemistry of the products were assigned on the basis of their spectroscopic analysis. For example, in the ^1^H-NMR spectrum of compound **4c**, a sharp singlet at *δ* 2.13 due to the N-methyl protons was seen. The benzylic proton exhibited a doublet of doublets at *δ* 3.71 (*J* = 10.4 Hz and 8.0 Hz). The off resonance decoupled ^13^C-NMR spectrum added conclusive support. The ^13^C-NMR spectrum of **5c** showed peaks at *δ* 81.17 and *δ* 67.26 for the two spirocarbons, respectively. The mass spectrum of **4c** showed a molecular ion peak at *m/z* 486.1392 (M+Na). The X-ray crystallographic study of single of **8b** (Figure 2) not only confirmed the structure deduced from NMR spectroscopic studies, but also determined the stereochemical outcome of the cycloaddition.

**Scheme 2:**
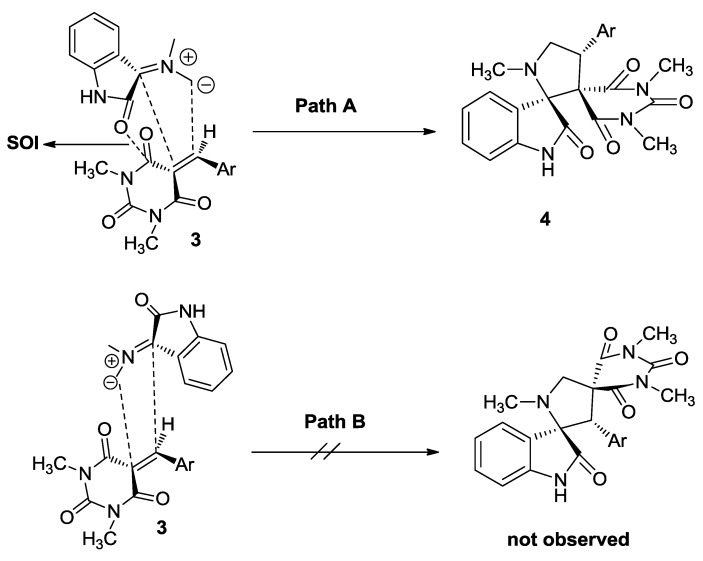
Proposed reaction mechanism for the formation of compound **4**.

Although the detailed mechanism of the above reaction has not been elucidated yet, the formation of **4** can be explained by the mechanism proposed in Scheme 2. The reaction proceeds through the generation of azomethine ylide (dipole **7**) via the condensation of isatin (**1**) with sarcosine (**2**) and decarboxylation. The dipolarophile **3** regioselectively reacts with azomethine ylides (dipole **7**) in ethanol to give the desired products dispiro compounds **4** (Scheme 2, path A). The cycloaddition proceeds via an *endo* transition state [39,40,41]. The regioselectivity in the product formation can be explained by considering the secondary orbital interaction (SOI) [42,43] of the orbital of the carbonyl group of dipolarophile **3** with those of the ylide **7** as shown in Scheme 2. In this transition state, these orbital interactions are typically orthogonal in nature and occur between the oxygen atom of the carbonyl of the isatin and the carbon atom of the carbonyl of the dipolarophile **3**. Accordingly, the observed regioisomer **4** via path A is more favorable because of the SOI which is not possible in path B.

## 3. Experimental

### 3.1. General

All reagents were purchased from commercial sources and used without further purification. Melting points are uncorrected. IR spectra were recorded on a Nicolet 6700 spectrometer in KBr with absorptions in cm^−1^. ^1^H-NMR spectra were determined on a Varian Inova-300/400 MHz spectrometer in DMSO-*d*_6_ solution. *J* values are in Hz. Chemical shifts are expressed in ppm downfield from internal standard TMS. HRMS data were obtained using Bruker micrOTOF-Q instrument or TOF-MS instrument. The starting compounds **3** were prepared according to the previously reported procedures [44,45].

### 3.2. General Procedure for the Synthesis of Dispirooxindoles ***4***, ***6*** and ***8***

A dry 50 mL flask was charged with isatin (**1**) or acenaphthenequinone (**7**) (0.5 mmol), sarcosine (**2**) or L-thioproline (**5**) (0.5 mmol), dipolarophile **3** (0.5 mmol) and ethanol (10 mL). The mixture was stirred at reflux temperature for 1–3 h. After completion of the reaction (monitored by TLC), the solvent was removed under vacuum. The solid was recrystallized from ethanol, and then dried at 80 °C for 4h under vacuum to give compounds **4**, **6** or **8**.

*2,7,9-Trimethyl-4-(4-bromophenyl)-1-(spiro-3'-indolino)-2,7,9-triazaspiro[4.5]decane-6,8,10-trione* (**4a**). White solid; m.p. 180–182 °C; IR (KBr, cm^−1^): 3313, 2939, 1735, 1679, 1618, 1468, 1420, 1374, 1070, 753; ^1^H-NMR (400 MHz, DMSO-*d*_6_): δ (ppm) 2.12 (s, 3H, CH_3_), 2.88–2.89 (m, 6H, 2 × CH_3_), 3.60 (m, 1H, CH_2_), 3.89 (t, *J* = 8.0 Hz, 1H, CH_2_), 5.18 (t, *J* = 8.8 Hz, 1H, CH), 6.78 (d, *J* = 7.6 Hz, 1H, ArH), 6.82 (d, *J* = 7.2 Hz, 1H, ArH), 6.96 (t, *J* = 6.4 Hz, 1H, ArH), 7.13–7.15 (m, 2H, ArH), 7.26 (t, *J* = 6.8 Hz, 1H, ArH), 7.41 (d, *J* = 8.4 Hz, 2H, ArH), 10.51 (s, 1H, NH); ^13^C-NMR (75 MHz, DMSO-*d*_6_): δ (ppm) 28.52, 29.67, 35.92, 41.98, 56.60, 67.26, 81.17, 110.42, 112.85, 120.15, 122.12, 123.59, 125.16, 131.02, 131.56, 137.96, 143.70, 150.33, 164.76, 166.92, 175.70; HRMS: calculated for C_23_H_21_^79^BrN_4_O_4_Na [M+Na]^+^: 519.0638, found: 519.0621.

*2,7,9-Trimethyl-4-(4-methylphenyl)-1-(spiro-3'-indolino)-2,7,9-triazaspiro[4.5]decane-6,8,10-trione* (**4b**). White solid; m.p. 190–191 °C; IR (KBr, cm^−1^): 3321, 2949, 1735, 1689, 1672, 1515, 1471, 1373, 752; ^1^H-NMR (400 MHz, DMSO-*d*_6_): δ (ppm) 2.13 (s, 3H, CH_3_), 2.23 (s, 3H, CH_3_), 2.90 (s, 6H, 2 × CH_3_), 3.56–3.60 (m, 1H, CH_2_), 3.91 (t, *J* = 9.2 Hz, 1H, CH_2_), 5.19 (t, *J* =9.2 Hz, 1H, CH), 6.76–6.78 (m, 1H, ArH), 6.82–6.84 (m, 1H, ArH), 6.96 (t, *J* = 7.2 Hz, 1H, ArH), 7.04 (s, 4H, ArH), 7.26 (t, *J* = 7.6 Hz, 1H, ArH), 10.49 (s, 1H, NH); ^13^C-NMR (100 MHz, DMSO-*d*_6_): δ (ppm) 21.17, 28.47, 29.61, 35.91, 42.26, 56.84, 67.33, 81.08, 110.36, 122.09, 123.73, 125.09, 128.43, 129.37, 131.63, 135.38, 135.93, 143.66, 150.33, 164.77, 167.02, 175.77; HRMS: calculated for C_24_H_24_N_4_O_4_[M]^+^: 432.1792, found: 432.1800.

*2,7,9-Trimethyl-4-(4-nitrophenyl)-1-(spiro-3'-indolino)-2,7,9-triazaspiro[4.5]decane-6,8,10-trione* (**4c**). White solid; m.p. 188–190 °C; IR (KBr, cm^−1^): 3350, 2926, 1730, 1684, 1619, 1599, 1520, 1469, 1379, 1348, 754; ^1^H-NMR (400 MHz, DMSO-*d*_6_): δ (ppm) 2.13 (s, 3H, CH_3_), 2.90 (s, 6H, 2 × CH_3_), 3.71 (dd, *J*_1_ = 8.0 Hz, *J*_2_ = 10.4 Hz, 1H, CH_2_), 3.93 (t, *J* = 8.0 Hz, 1H, CH_2_), 5.30 (t, *J* = 9.2 Hz, 1H, CH), 6.78–6.84 (m, 2H, ArH), 6.97 (t, *J* = 7.6 Hz, 1H, ArH), 7.28 (t, *J* = 8.0 Hz, 1H, ArH), 7.44 (d, *J* = 8.8 Hz, 2H, ArH), 8.08 (d, *J* = 8.8 Hz, 2H, ArH), 10.57 (s, 1H, NH); ^13^C-NMR (75 MHz, DMSO-*d*_6_): δ (ppm) 30.51, 31.670, 37.85, 44.31, 58.50, 69.16, 83.07, 112.41, 124.11, 125.34, 125.67, 127.20, 131.77, 133.77, 145.70, 148.45, 148.98, 152.31, 166.77, 168.80, 177.57; HRMS: calculated for C_23_H_21_N_5_O_6_Na [M+Na]^+^: 486.1384, found: 486.1392.

*2,7,9-Trimethyl-4-(4-chlorophenyl)-1-(spiro-3'-indolino)-2,7,9-triazaspiro[4.5]decane-6,8,10-trione* (**4d**). White solid; m.p. 240–242 °C; IR (KBr, cm^−1^): 3317, 2926, 1736, 1718, 1679, 1620, 1570, 1468, 1379, 758; ^1^H-NMR (400 MHz, DMSO-*d*_6_): δ (ppm) 2.89 (s, 3H, CH_3_), 2.90 (s, 3H, CH_3_), 3.08 (s, 3H, CH_3_), 3.61 (dd, *J*_1_ = 8.4 Hz, *J*_2_ = 10.0 Hz, 1H, CH_2_), 3.89 (t, *J* = 8.0 Hz, 1H, CH_2_), 5.20 (t, *J* = 9.2 Hz, 1H, CH), 6.78 (d, *J* = 8.0 Hz, 1H, ArH), 6.82–6.84 (m, 1H, ArH), 6.96 (t, *J* = 7.6 Hz, 1H, ArH), 7.19–7.21 (m, 1H, ArH), 7.29 (d, *J* = 8.4 Hz, 3H, ArH), 7.33–7.35 (m, 1H, ArH), 10.52 (s, 1H, NH); ^13^C-NMR (100 MHz, DMSO-*d*_6_): δ (ppm) 28.53, 29.67, 35.93, 41.97, 56.69, 67.34, 81.18, 110.42, 122.13, 123.62, 125.17, 128.66, 130.63, 131.63, 131.72, 137.55, 143.72, 150.53, 164.78, 166.94, 175.70; HRMS: calculated for C_23_H_21_^35^ClN_4_O_4_ [M]^+^: 452.1251, found: 452.1260. 

*1,3-Dimethyl-5'-(4-bromophenyl)-7'-(spiro-3''-indolino)tetrahydro-1H,1'H-spiro[pyrimidine-5,6'-pyrrolo[1,2-c]thiazole]-2,4,6-trione* (**6a**). White solid; m.p. 194–196 °C; IR (KBr, cm^−1^): 3236, 2926, 1741, 1719, 1679, 1615, 1469, 1376, 749; ^1^H-NMR (400 MHz, DMSO-d_6_): δ (ppm) 2.94 (s, 3H, CH_3_), 3.05–3.08 (m, 1H, CH_2_), 3.28–3.33 (m, 2H, CH_2_), 3.40–3.42 (m, 4H, CH_3_ and CH), 3.79 (d, *J* = 10.4 Hz, CH), 4.29 (d, *J* = 10.0 Hz, 1H, CH_2_), 4.96–5.00 (m, 1H, CH_2_), 6.82 (d, *J* = 7.2 Hz, 1H, ArH), 6.98–7.02 (m, 1H, ArH), 7.29 (t, *J* = 7.2 Hz, 1H, ArH), 7.39–7.41 (m, 2H, ArH), 7.46–7.48 (m, 2H, ArH), 7.61 (d, *J* = 7.2 Hz, 1H, ArH), 10.84 (s, 1H, NH); ^13^C-NMR (75 MHz, DMSO-d_6_): δ (ppm) 33.93, 34.09, 42.24, 55.22, 58.79, 76.38, 76.57, 85.45, 115.22, 125.77, 126.48, 127.08, 135.10, 136.20, 136.30, 137.28, 140.40, 147.07, 155.71, 169.89, 171.56, 180.61; HRMS: calculated for C_24_H_22_^79^BrN_4_O_4_S [M+H]^+^: 541.0540, found: 541.0559.

*1,3-Dimethyl-5'-(4-methylphenyl)-7'-(spiro-3''-indolino)tetrahydro-1H,1'H-spiro[pyrimidine-5,6'-pyrrolo[1,2-c]thiazole]-2,4,6-trione* (**6b)**. White solid; m.p. 188–190 °C; IR (KBr, cm^−1^): 3213, 2921, 1740, 1684, 1620, 1472, 1369, 752; ^1^H-NMR (400 MHz, DMSO-d_6_): δ (ppm) 2.24 (s, 3H, CH_3_), 2.52 (s, 3H, CH_3_), 2.95 (s, 3H, CH_3_), 3.02 (dd, *J*_1_ = 3.2 Hz, *J*_2_ = 11.2 Hz, 1H, CH_2_), 3.31–3.33 (m, 1H, CH_2_), 3.38–3.40 (m, 1H, CH), 3.78–3.81 (m, 1H, CH), 4.28–4.31 (m, 1H, CH_2_), 4.98–5.03 (m, 1H, CH_2_), 6.82 (d, *J* = 7.6 Hz, 1H, ArH), 7.00 (t, *J* = 7.6 Hz, 1H, ArH), 7.07–7.09 (m, 2H, ArH), 7.27–7.32 (m, 3H, ArH), 7.61 (d, *J* = 7.6 Hz, 1H, ArH), 10.76 (s, 1H, NH); ^13^C-NMR (75 MHz, DMSO-d_6_): δ (ppm) 25.95, 33.90, 34.10, 42.45, 55.63, 58.78, 76.57, 76.63, 85.40, 115.26, 126.45, 127.13, 134.11, 134.72, 135.12, 136.13, 137.83, 141.50, 147.05, 155.68, 169.81, 171.56, 180.65; HRMS: calculated for C_25_H_25_N_4_ O_4_S [M]^+^: 477.1591, found: 477.1607. 

*1,3-Dimethyl-5'-(4-nitrophenyl)-7'-(spiro-3''-indolino)tetrahydro-1H,1'H-spiro[pyrimidine-5,6'-pyrrolo[1,2-c]thiazole]-2,4,6-trione* (**6c**). White solid; m.p. 186–188 °C; IR (KBr, cm^−1^): 3205, 3086, 2952, 1741, 1683, 1522, 1419, 1348, 749; ^1^H-NMR (400 MHz, DMSO-d_6_): δ (ppm) 2.94 (s, 3H, CH_3_), 3.14–3.17 (m, 2H, CH_2_), 3.41 (s, 4H, CH_3_ and CH), 3.79 (d, *J* = 10.0 Hz, 1H, CH), 4.46–4.48 (m, 1H, CH_2_), 5.02 (s, 1H, CH_2_), 6.82–6.84 (m, 1H, ArH), 7.00–7.02 (m, 1H, ArH), 7.28–7.30 (m, 1H, ArH), 7.61–7.62 (m, 1H, ArH), 7.68–7.70 (m, 2H, ArH), 8.11–8.13 (m, 2H, ArH), 10.86 (s, 1H, NH); ^13^C-NMR (100 MHz, DMSO-d_6_): δ (ppm) 29.21, 29.39, 37.49, 50.57, 53.77, 71.59, 72.13, 80.60, 121.79, 122.27, 123.58, 130.29, 131.38, 131.53, 142.33, 144.55, 146.96, 150.95, 165.20, 166.83, 175.71; HRMS: calculated for C_24_H_22_N_5_O_6_S [M+H]^+^: 508.1285, found: 508.1290. 

*1,3-Dimethyl-5'-(4-chlorophenyl)-7'-(spiro-3''-indolino)tetrahydro-1H,1'H-spiro[pyrimidine-5,6'-pyrrolo[1,2-c]thiazole]-2,4,6-trione* (**6d**). White solid; m.p. 162–164 °C; IR (KBr, cm^−1^): 3289, 3062, 2908, 1751, 1733, 1680, 1496, 1376, 755; ^1^H-NMR (400 MHz, DMSO-d_6_): δ (ppm) 2.87 (s, 3H, CH_3_), 2.89 (s, 3H, CH_3_), 3.18 (t, *J* = 9.6 Hz, 1H, CH_2_), 3.25–3.29 (m, 1H, CH_2_), 3.42 (d, *J* = 7.6 Hz, 1H, CH), 3.72 (d, *J* = 7.6 Hz, 1H, CH), 4.70–4.74 (m, 1H, CH_2_), 4.83–4.85 (m, 1H, CH_2_), 6.77–6.79 (m, 1H, ArH), 6.94–7.01 (m, 2H, ArH), 7.25–7.29 (m, 1H, ArH), 7.32–7.34 (m, 4H, ArH), 10.71 (s, 1H, NH); HRMS: calculated for C_24_H_21_^35^ClN_4_O_4_SNa [M+Na]^+^: 519.0864, found: 519.0871. 

*1,3-Dimethyl-5'-phenyl-7'-(spiro-3''-indolino)tetrahydro-1H,1'H-spiro[pyrimidine-5,6'-pyrrolo[1,2-c]thiazole]-2,4,6-trione* (**6e**). White solid; m.p. 183–184 °C; IR (KBr, cm^−1^): 3212, 3082, 2953, 1740, 1680, 1615, 1472, 1367, 754; ^1^H-NMR (400 MHz, DMSO-*d*_6_): δ (ppm) 2.53 (s, 3H, CH_3_), 2.96 (s, 3H, CH_3_), 3.07 (dd, *J*_1_ = 3.6 Hz, *J*_2_ = 11.2 Hz, 1H, CH_2_), 3.33–3.35 (m, 1H, CH_2_), 3.39 (d, *J* = 10.0 Hz, 1H, CH), 3.80 (d, *J* = 10.4 Hz, 1H, CH), 4.36 (d, *J* = 10.0 Hz, 1H, CH_2_), 4.99–5.04 (m, 1H, CH_2_), 6.83 (d, *J* = 7.6 Hz, 1H, ArH), 7.01 (t, *J* = 7.6 Hz, 1H, ArH), 7.19–7.23 (m, 1H, ArH), 7.26–7.31 (m, 3H, ArH), 7.40–7.42 (m, 2H, ArH), 7.61 (d, *J* = 7.6 Hz, 1H, ArH), 10.77 (s, 1H, NH); ^13^C-NMR (100 MHz, DMSO-*d*_6_): δ (ppm) 34.27, 34.46, 42.90, 56.16, 59.00, 76.97, 85.73, 115.54, 126.83, 127.51, 132.64, 133.88, 135.04, 135.50, 136.49, 141.47, 147.38, 156.06, 170.16, 171.94, 180.97; HRMS: calculated for C_24_H_23_N_4_O_4_S [M+H]^+^: 463.1435, found: 463.1443. 

*1,3-Dimethyl-5'-(2-nitrophenyl)-7'-(spiro-3''-indolino)tetrahydro-1H,1'H-spiro[pyrimidine-5,6'-pyrrolo[1,2-c]thiazole]-2,4,6-trione* (**6f**). White solid; m.p. 184–186 °C; IR (KBr, cm^−1^): 3220, 2947, 1743, 1685, 1616, 1536, 1471, 1370, 782, 763; ^1^H-NMR (400 MHz, DMSO-d_6_): δ (ppm) 2.34 (s, 3H, CH_3_), 2.97 (s, 3H, CH_3_), 3.00 (s, 1H, CH_2_), 3.04 (s, 1H, CH_2_), 3.10–3.14 (m, 1H, CH), 3.82 (d, *J* =10.8 Hz, 1H, CH), 4.88–4.90 (m, 1H, CH_2_), 5.07–5.11 (m, 1H, CH_2_), 6.81–6.83 (m, 1H, ArH), 7.01 (t, *J* = 7.6 Hz, 1H, ArH), 7.29 (t, *J* =7.6 Hz, 1H, ArH), 7.50 (t, *J* =8.0 Hz, 1H, ArH), 7.59 (d, *J* = 7.6 Hz, 1H, ArH), 7.68 (t, *J* = 8.0 Hz, 1H, ArH), 7.78 (d, *J* = 7.6 Hz, 1H, ArH), 8.36 (d, *J* = 8.0 Hz, 1H, ArH), 10.84 (s, 1H, NH); HRMS: calculated for C_24_H_22_N_5_O_6_S [M+H]^+^: 508.1285, found: 508.1296. 

*1,3-Dimethyl-5'-(3,4-dichlorophenyl)-7'-(spiro-3''-indolino)tetrahydro-1H,1'H-spiro[pyrimidine-5,6'-pyrrolo[1,2-c]thiazole]-2,4,6-trione* (**6g**). White solid; m.p. 192–194 °C; IR (KBr, cm^−1^): 3074, 2957, 1757, 1721, 1688, 1614, 1469, 1367, 748; ^1^H-NMR (400 MHz, DMSO-*d*_6_): δ (ppm) 2.46 (s, 3H, CH_3_), 2.94 (s, 3H, CH_3_), 3.03 (dd, *J*_1_ = 2.4 Hz, *J*_2_ = 11.2 Hz, 1H, CH_2_), 3.23–3.28 (m, 1H, CH_2_), 3.40 (s, 1H, CH), 3.78 (d, *J* = 10.8 Hz, 1H, CH), 4.27 (d, *J* = 10.0 Hz, 1H, CH_2_), 4.99–5.04 (m, 1H, CH_2_), 6.83 (d, *J* = 7.6 Hz, 1H, ArH), 7.00 (t, *J* = 7.6 Hz, 1H, ArH), 7.30 (t, *J* = 7.6 Hz, 1H, ArH), 7.49–7.56 (m, 2H, ArH), 7.63 (d, *J* = 7.6 Hz, 1H, ArH), 7.71 (s, 1H, ArH), 10.84 (s, 1H, NH); ^13^C-NMR (75 MHz, DMSO-*d*_6_): δ (ppm) 28.69, 35.78, 43.32, 57.18, 67.90, 84.56, 120.35, 122.04, 122.14, 127.39, 128.96, 129.47, 130.19, 130.47, 130.92, 131.70, 132.97, 133.13, 137.67, 142.05, 149.92, 164.77, 167.07, 203.16; HRMS: calculated for C_24_H_21_^35^Cl_2_N_4_O_4_S [M+H]^+^: 531.0655, found: 531.0674. 

*1,3-Dimethyl-5'-(thiophen-2-yl)-7'-(spiro-3''-indolino)tetrahydro-1H,1'H-spiro[pyrimidine-5,6'-pyrrolo[1,2-c]thiazole]-2,4,6-trione* (**6h**). White solid; m.p. 164–166 °C; IR (KBr, cm^−1^): 3243, 3078, 2956, 1738, 1679, 1568, 1439, 1382; ^1^H-NMR (400 MHz, DMSO-*d*_6_): δ (ppm) 2.88 (s, 3H, CH_3_), 2.92 (s, 3H, CH_3_), 3.12–3.17 (m, 2H, CH_2_), 3.44 (d, *J* = 7.6 Hz, 1H, CH), 3.70 (d, *J* = 7.2 Hz, 1H, CH), 4.72–4.76 (m, 1H, CH_2_), 5.05 (d, *J* = 8.4 Hz, 1H, CH_2_), 6.79 (d, *J* = 7.6 Hz, 1H, ArH), 6.92–7.00 (m, 4H, ArH), 7.25–7.29 (m, 1H, ArH), 7.37–7.38 (m, 1H, ArH), 10.74 (s, 1H, NH); ^13^C-NMR (75 MHz, DMSO-*d*_6_): δ (ppm) 28.68, 29.70, 36.56, 43.83, 51.33, 73.06, 74.23, 79.44, 110.74, 122.54, 124.32, 125.06, 126.02, 127.49, 127.69, 132.03, 138.48, 142.86, 150.04, 164.75, 165.76, 176.46; HRMS: calculated for C_22_H_21_N_4_O_4_S_2_ [M+H]^+^: 469.0999, found: 469.0980. 

*2,7,9-Trimethyl-4-phenyl-1-(spiro-2'-acenaphthylenone)-2,7,9-triazaspiro[4.5]decane-6,8,10-trione* (**8a**). White solid; m.p. 168–170 °C; IR (KBr, cm^−1^): 3063, 2920, 1748, 1723, 1685, 1442, 1371, 833, 783, 751; ^1^H-NMR (400 MHz, DMSO-*d*_6_): δ (ppm) 2.19 (s, 6H, 2 × CH_3_), 2.91 (s, 3H, CH_3_), 3.83 (t, *J* = 8.0 Hz, 1H, CH_2_), 4.08 (t, *J* = 8.0 Hz, 1H, CH_2_), 5.18 (t, *J* = 8.0 Hz, 1H, CH), 7.16 (s, 3H, ArH), 7.25 (s, 2H, ArH), 7.30 (d, *J* = 8.0 Hz, 1H, ArH), 7.72 (s, 1H, ArH), 7.82–7.88 (m, 2H, ArH), 8.04–8.06 (m, 1H, ArH), 8.29–8.31 (m, 1H, ArH); ^13^C-NMR (100 MHz, DMSO-*d*_6_): δ (ppm) 33.40, 33.45, 40.51, 48.79, 62.13, 72.64, 89.12, 126.78, 126.80, 131.85, 132.07, 133.12, 133.68, 133.70, 134.19, 135.06, 135.22, 137.60, 138.01, 143.03, 146.74, 154.67, 169.55, 171.95, 207.93; HRMS: calculated for C_27_H_23_N_3_O_4_Na [M+Na]^+^: 476.1581, found: 476.1582.

*2,7,9-Trimethyl-4-(3,4-dichlorophenyl)-1-(spiro-2'-acenaphthylenone)-2,7,9-triazaspiro[4.5]decane-6,8,10-trione* (**8b**). White solid; m.p. 192–194 °C; IR (KBr, cm^−1^): 2965, 1721, 1684, 1640, 1372, 783, 750; ^1^H-NMR (400 MHz, DMSO-*d*_6_): δ (ppm) 2.12 (s, 3H, CH_3_), 2.17 (s, 3H, CH_3_), 2.89 (s, 3H, CH_3_), 3.79 (t, *J* = 8.0 Hz, 1H, CH_2_), 4.05 (t, *J* = 8.0 Hz, 1H, CH_2_), 5.15 (t, *J* = 7.6 Hz, 1H, CH), 7.17–7.19 (m, 1H, ArH), 7.28–7.30 (m, 1H, ArH), 7.46–7.51 (m, 2H, ArH), 7.71–7.75 (m, 1H, ArH), 7.83–7.90 (m, 2H, ArH), 8.07 (d, *J* = 8.0 Hz, 1H, ArH), 8.32 (d, *J* = 7.2 Hz, 1H, ArH); ^13^C-NMR (75 MHz, DMSO-*d*_6_): δ (ppm) 28.66, 35.73, 42.82, 56.93, 56.95, 68.00, 84.70, 120.77, 122.08, 122.15, 127.42, 128.89, 129.44, 129.99, 130.07, 130.42, 130.70, 131.09, 131.22, 131.39, 133.01, 139.12, 142.07, 149.89, 164.73, 166.92, 203.09; HRMS: calculated for C_27_H_21_Cl_2_N_3_O_4_Na [M+Na]^+^: 509.1113, found: 509.1338.

*2,7,9-Trimethyl-4-(3,4-methylenedioxylphenyl)-1-(spiro-2'-acenaphthylenone)-2,7,9-triazaspiro[4.5]decane-6,8,10-trione* (**8c**). White solid; m.p. 186–188 °C; IR (KBr, cm^−1^): 2939, 2897, 1730, 1692, 1500, 1364, 1233, 832, 783; ^1^H-NMR (400 MHz, DMSO-*d*_6_): δ (ppm) 2.16 (s, 6H, 2 × CH_3_), 2.91 (s, 3H, CH_3_), 3.73 (s, 1H, CH_2_), 4.02 (s, 1H, CH_2_), 5.10 (s, 1H, CH), 5.96 (s, 2H, CH_2_), 6.64 (s, 1H, ArH), 6.77–6.82 (m, 2H, ArH), 7.30 (s, 1H, ArH), 7.72–7.87 (m, 3H, ArH), 8.05 (s, 1H, ArH), 8.30 (s, 1H, ArH); ^13^C-NMR (75 MHz, DMSO-*d*_6_): δ (ppm) 27.39, 34.47, 42.72, 56.19, 67.02, 83.22, 100.23, 107.34, 108.13, 120.73, 120.89, 126.02, 127.68, 128.15, 129.06, 129.20, 130.11, 131.55, 132.06, 140.67, 145.25, 146.47, 148.62, 163.47, 165.86, 201.87; HRMS: calculated for C_28_H_23_N_3_O_6_ [M]^+^: 497.1587, found: 497.1585.

### 3.3. X-ray Crystallography [46]

The single-crystals of compound **8b** were obtained by slow evaporation from ethanol. Intensity data were collected on a Bruker P4 diffractometer with graphite monochromated Mo K*α* radiation (*λ* = 0.71073 Å) using the *ω* scan mode with 1.34º < *θ* < 25.02º; 4188 unique reflections were measured and 3254 reflections with *I* > 2*σ*(*I*) were used in the Fourier techniques. The final refinement was converged to *R* = 0.0428 and *wR* = 0.1266. Crystal data for **8b**: empirical formula C_27_H_21_Cl_2_N_3_O_4_, crystal dimension 0.42 × 0.40 × 0.37 mm, triclinic, space group P-1, *a* = 8.0847(7) Å, *b* = 10.0554(10) Å, *c* = 15.5500(13) Å, *α* = 76.8210(10)º, *β* = 86.626(2)º, *γ* = 76.2960(10)º, V = 1195.79(19)Å^3^, *M*r = 522.37, Z = 2, *D*c = 1.451 Mg/m^3^, *μ*(Mo K*α*) = 0.312 mm^−1^, *F*(000) = 540, *S* = 1.079.

## 4. Conclusions

In summary, we have successfully developed a 1,3-dipolar cycloaddition of azomethine ylides, and a series of novel dispiro cycloadducts were obtained. This method has the advantages of convenient operation, mild reaction conditions, short reaction time, and high efficiency.
